# Historical and contemporary hypotheses on the development of oral diseases: are we there yet?

**DOI:** 10.3389/fcimb.2014.00092

**Published:** 2014-07-16

**Authors:** Bob T. Rosier, Marko De Jager, Egija Zaura, Bastiaan P. Krom

**Affiliations:** ^1^Department of Preventive Dentistry, Academic Centre for Dentistry Amsterdam (ACTA), University of Amsterdam and Free University AmsterdamAmsterdam, Netherlands; ^2^Philips ResearchEindhoven, Netherlands

**Keywords:** dental plaque, ecological plaque hypothesis, keystone pathogen hypothesis, specific plaque hypothesis, non-specific plaque hypothesis

## Abstract

Dental plaque is an oral biofilm that much like the rest of our microbiome has a role in health and disease. Specifically, it is the cause of very common oral diseases such as caries, gingivitis, and periodontitis. The ideas about oral disease development have evolved over time. In the nineteenth century, scientists could not identify bacteria related to disease due to the lack of technology. This led to the “Non-Specific Plaque Hypothesis” or the idea that the accumulation of dental plaque was responsible for oral disease without discriminating between the levels of virulence of bacteria. In the twentieth century this idea evolved with the techniques to analyze the changes from health to disease. The first common hypothesis was the “Specific Plaque Hypothesis” (1976) proposing that only a few species of the total microflora are actively involved in disease. Secondly, the “Non-Specific Plaque Hypothesis” was updated (1986) and the idea that the overall activity of the total microflora could lead to disease, was enriched by taking into account difference in virulence among bacteria. Then, a hypothesis was considered that combines key concepts of the earlier two hypotheses: the “Ecological Plaque Hypothesis” (1994), which proposes that disease is the result of an imbalance in the microflora by ecological stress resulting in an enrichment of certain disease-related micro-organisms. Finally, the recent “Keystone-Pathogen Hypothesis” (2012) proposes that certain low-abundance microbial pathogens can cause inflammatory disease by interfering with the host immune system and remodeling the microbiota. In this comprehensive review, we describe how these different hypotheses, and the ideas around them, arose and test their current applicability to the understanding of the development of oral disease. Finally, we conclude that an all-encompassing ecological hypothesis explaining the shifts from health to disease is still lacking.

## Introduction

The human body contains 10 times more bacterial cells than human cells (Turnbaugh et al., 2007), with hundreds of times more bacterial than human genes (Yang et al., 2009). This microbiome has a significant influence on the physical and mental well-being of the host (Wikoff et al., 2009; Archambaud et al., 2013) and interactions between the microbiome and the host dictate health and disease.

The mouth is a nutrition-rich, humid environment of around 35/36°C with a resting-pH between 6.75 and 7.25 (Marsh, 2003; De Almeida et al., 2008). These conditions are optimal for growth of many bacteria that can form biofilms—a structured, often polymicrobial community—on oral surfaces (Jenkinson and Lappin-Scott, 2001). Oral biofilms, called dental plaque, were first observed by Antoni van Leeuwenkoek in the seventeenth century (van Leeuwenhoek, 1684) and are associated with all of the most common oral diseases: caries, and periodontal disease.

Poor oral health has been linked to many systemic diseases, including cardiovascular disease, diabetes, adverse pregnancy outcomes (Seymour et al., 2007), rheumatoid arthritis (Mercado et al., 2001), gastrointestinal disease (Watabe et al., 1998), oral cancer (Tezal et al., 2009), and pre-eclampsia (Kumar et al., 2014). Furthermore, the two most common oral diseases, caries and periodontal disease, are highly abundant among the population of industrialized countries, having a major impact on the populations' well-being and health care providers (Petersen and Lennon, 2004). To effectively treat and prevent these oral diseases, it is important to understand how healthy plaque develops into pathological plaque.

The ideas about how changes in dental plaque relate to a shift from oral health to disease have changed over time. In this review we discuss the main hypotheses of oral disease development that were proposed between the nineteenth and the twenty first century: the Traditional and Updated Non-Specific Plaque Hypothesis (NSPH), the Specific Plaque Hypothesis (SPH), the Ecological Plaque Hypothesis (EPH) and the Keystone Pathogen Hypothesis (KPH) (Table 1). We then test their current applicability to the understanding of the development of oral disease.

**Table 1:** **Comparison of the different hypotheses**.

**Hypothesis**	**References**	**Bacteria involved in disease**	**Relates to**	
			**Ecological changes**	Host specific factors^*^
Traditional NSPH	Miller, 1890	All	−	−
SPH	Loesche, 1976	Specific bacteria	−	−
Updated NSPH	Theilade, 1986	All, difference in virulence	+	−
EPH	Marsh, 1994	All, enrichment of specific pathogenic bacteria	+++	−
KPH	Hajishengallis et al., 2012	Specific bacteria, dependent on (some of) remaining microbiota	++	+

* Factors that could differ amongst hosts, e.g., innate immune system (levels of cytokine and TLR expression), response to certain bacteria, GCF properties (iron concentration), saliva properties (buffer capacity) and enamel repair. − not or only briefly mentioned, + mentioned, ++ mentioned and described, +++ described in detail.

## Traditional non-specific plaque hypothesis (T-NSPH)

The NSPH are part of a controversy that took place for over a century (Miller, 1890; Loesche, 1976; Theilade, 1986). At the end of the nineteenth century the most common idea about dental infections was that they were caused by the non-specific overgrowth of all bacteria in dental plaque (Black, 1884, 1899; Miller, 1890; Loesche, 1986). This idea is referred to as the “Non-specific plaque hypothesis” (NSPH) (Loesche, 1976) and was based on work of researchers such as Black (1884) and Miller (1890). Applying the NSPH it was postulated that it was the quantity of plaque that determined the pathogenicity without discriminating between the levels of virulence of bacteria. Believing this, the host would have a threshold capacity to detoxify bacterial products (e.g., saliva neutralizing acid) and disease would only develop if this threshold was surpassed and the virulence factors could no longer be neutralized (Theilade, 1986). The conclusion was that if any plaque has an equal potential to cause disease, the best way of disease prevention would be non-specific mechanical removal of as much plaque as possible by e.g., tooth brushing or tooth picking. The improvement of techniques to isolate and identify bacteria in the mid-20th century led to the abandoning of the NSPH. Nonetheless, mechanical plaque removal remained the most efficient way of preventing disease.

## The specific plaque hypothesis (SPH)

In the 1970s, culture-based techniques and microscopy allowed discrimination of specific bacterial species and opened the hunt for disease-related micro-organisms. It was noticed that the antibiotic kanamycin was particularly effective against cariogenic species such as oral streptococci and reduced caries formation (Loesche and Nafe, 1973; Loesche et al., 1977). This suggested that removing cariogenic bacteria from the oral cavity using antibiotics could prevent caries. In 1976, Walter J. Loesche announced the “Specific Plaque Hypothesis” (SPH), postulating that dental caries was an infection with specific bacteria in the dental plaque of which the most relevant were “mutans streptococci” (main species: *Streptococcus mutans* and *Streptococcus sobrinus*) and lactobacilli (Loesche, 1976).

This hypothesis proposed that use of antibiotics against specific bacterial species could cure and prevent caries (Loesche and Nafe, 1973; Loesche, 1976, 1986; Loesche et al., 1977). However, results from clinical studies, then and today, are not very promising. For instance, even though the use of kanamycin resulted in an overall reduction of caries, at some surfaces the caries rate increased. This indicates that kanamycin failed to penetrate certain niches allowing cariogenic species to have a selective advantage and accumulate there (Loesche et al., 1977; Banas, 2009). Furthermore antibiotics reduced the abundance of cariogenic bacteria but failed to eliminate them thus as soon as the treatment was stopped, abundance increased (Loesche and Nafe, 1973; Loesche et al., 1977), while a long period of treatment leads to antibiotic resistance (Kornman and Karl, 1982). These suggested “specific-pathogens” are part of the indigenous microflora and unlike foreign pathogens cannot be eliminated from the oral cavity (van Palenstein Helderman, 1984).

The development of the anaerobic hood in the 1970s for the first time allowed cultivation of the strict anaerobic species. This extended the SPH to periodontal diseases which were proposed to be inflammations caused by specific periopathogens and antibiotic treatment would be effective (Loesche, 1986). However, in line with the use of antibiotics in caries treatment, recent clinical studies evaluating the effectiveness of antibiotics as adjunct in periodontal therapy have not booked significant success either. A Cochrane review stated that the use of the antibiotic chlorhexidine after scaling and root planing in patients with chronic periodontitis had only a modest positive effect, and concluded that the extensive use of chlorhexidine may be questioned (Eberhard et al., 2008).

In the decade after the SPH was introduced, potential periopathogens included: protozoa, spirochetes, streptococci, and actinomyces. In addition, Gram-negative, anaerobic rods including black-pigmented *Bacteriodes* such as *Bacteriodes melaninogenicus* (renamed to *Prevotela melaninogenica*) and others from the genus *Wolinella* (re-classified as *Campylobacter*) and facultative anaerobic, Gram-negative rods of the genera *Capnocytophaga, Eikenella* and *Actinobacillus* (van Palenstein Helderman, 1981; Socransky et al., 1982; Slots and Genco, 1984 and reviewed by Theilade, 1986) were identified as periopathogens. However, these findings were limited due to the large number of uncultivable species (~50%) (Siqueira and Rôças, 2013) and the bias toward easily cultivable species (Handelsman, 2004). The finding of different species related to periodontal disease led to the idea that oral disease could be initiated by a number of specific pathogens (Socransky, 1977; Theilade, 1986). This idea was further investigated over the next decades and led to the famous Socransky-complexes which include bacterial clusters based on their association with periodontal disease (Socransky et al., 1998).

## Updated non-specific plaque hypothesis (U-NSPH)

Else Theilade also noticed that the “specific-pathogens” from the SPH were indigenous bacteria and sometimes common bacteria in health, which led to an updated NSPH in 1986 focusing on periodontal disease (Theilade, 1986). At this time most researchers seemed to agree that gingivitis was a non-specific inflammatory reaction to a complex indigenous microbiota. However, the updated NSPH took into consideration that some indigenous subgingival bacteria can be more virulent than others and that plaque composition changes from health to disease. Nevertheless, it stated that all bacteria in plaque contribute to the virulence of the microflora by having a role in either colonization, evasion of the defense mechanism, and/or provocation of inflammation and tissue destruction (Theilade, 1986). Theilade's statement that “any microbial colonization of sufficient quantity in the gingival crevice causes at least gingivitis” was supported by the fact that a non-pathogenic plaque (i.e., not causing gingivitis in the absence of oral hygiene) had never been observed. Additionally, it was considered that some people have gingivitis for a lifetime without tissue and bone destruction, while others encounter rapid progression into periodontitis. Unlike the classic NSPH, the updated NSPH could explain this by taking into account that differences in the plaque microbial composition could lead to differences in pathogenic potential.

## Ecological plaque hypothesis (EPH)

In 1994 Philip D. Marsh proposed a hypothesis that combined key concepts of the earlier hypotheses. In his “Ecological Plaque Hypothesis” (EPH), disease is the result of an imbalance in the total microflora due to ecological stress, resulting in an enrichment of some “oral pathogens” or disease-related micro-organisms (Marsh, 1994). This idea was not entirely new since Theilade, in the review proposing the U-NSPH concluded that “increased virulence of plaque (leading to disease) is due to a plaque ecology unfavorable to the host and favorable for overgrowth by some of the indigenous bacteria having a pathogenic potential” (Theilade, 1986). Importantly, Marsh expanded this theory and related the changes in microbial composition to changes in ecological factors such as the presence of nutrients and essential cofactors, pH and redox potential (Marsh, 1994, 2003). For example, frequent exposure to a low pH, for instance as the result of sugar fermentation, leads to a relative increase of acid-tolerant species. The thought arose that disease could be prevented by interfering with processes that break down homeostasis and change composition. For example, non-fermentable sweeteners could be used to replace sugar and thus prevent acidification.

Marsh provided and collected convincing evidence to support his hypothesis, and it is still generally accepted that the composition of dental plaque depends on the environment. Thus, the classical “everything is everywhere, but, the environment selects” (Baas Becking, 1934) was successfully applied to dentistry (Marsh, 2003; De Wit and Bouvier, 2006). Marsh also considered the reverse: the bacteria in dental plaque affect the environment. For instance, early colonizers of supragingival dental surfaces, are usually facultative anaerobic bacteria that use the oxygen, producing carbon dioxide and hydrogen (Alexander, 1971; Marsh, 2003). This lowers the redox potential giving strict anaerobes a chance to settle and multiply in the biofilm. Bacterial growth is dictated by the environment, which in turn is influenced by bacterial metabolism, leading to mutual dependencies in health but also a chain of events that lead to diseases.

The importance of the host-dependent environment in selection of bacterial species that colonize should not be neglected. A simple but convincing example is a study indicating that, even though there is a continuous passage of bacteria from saliva to the gut, only 29 out of over 500 taxa found in the mouth were recovered in fecal samples (Moore and Moore, 1994). However, like the other hypotheses, the traditional EPH does not address the role of genetic factors of the host that significantly contribute to the composition of dental plaque and to susceptibility to disease (Mason et al., 2013).

## Keystone pathogen hypothesis (KPH)

The concept of keystone species is derived from basic ecological studies. Certain species have an effect on their environment that is *disproportional* relative to their overall abundance (Paine, 1969; Power et al., 1996; Darveau et al., 2012). George Hajishengallis and colleagues applied this concept to (oral) microbiology by proposing “The Keystone-Pathogen Hypothesis” (KPH) (Hajishengallis et al., 2012). The KPH indicates that certain low-abundance microbial pathogens can cause inflammatory disease by increasing the quantity of the normal microbiota and by changing its composition (Hajishengallis et al., 2012). For instance, *Porphyromonas gingivalis* is shown to be able to manipulate the native immune system of the host (reviewed by Darveau, 2010). By doing so it was hypothesized that it does not only facilitate its own survival and multiplication, but of the entire microbial community. In contrast to dominant species that can influence inflammation by their abundant presence, keystone pathogens can trigger inflammation when they are present in low numbers (Hajishengallis et al., 2012). When disease develops and advanced stages are reached, the keystone pathogen are detected in higher numbers (Socransky et al., 1998). Importantly, even though their absolute number increases, keystone pathogens can decrease in levels compared to the total bacterial load which increases as plaque accumulates in periodontitis (Abusleme et al., 2013).

The KPH was developed by observing the properties of the “red complex” (Socransky et al., 1998) bacterium *P. gingivalis*. Studies in mouse models showed that very low presence of *P. gingivalis* (<0.01% of the total bacterial count in plaque) could alter the plaque composition, leading to periodontitis (Hajishengallis et al., 2011). In germ-free mice, *P. gingivalis* was able to colonize by itself, but was not able to trigger disease without the presence of other bacterial species. This indicates that (some of) the commensal microbiota is essential in the disease process. Evidence of *P. gingivalis* acting as a keystone pathogen was also obtained in rabbit models (Hasturk et al., 2007) and non-human primates (Page et al., 2007).

The role of the host-immune system is critical in the KPH. At health, periodontal tissue contains a wall of neutrophils, between the plaque and the epithelial surface, residing just outside the epithelial cells. Expression of mediators such as interleukin 8 (IL-8), intercellular adhesion molecule (ICAM) and E-selectin is required to form this neutrophil wall (Moughal et al., 1992; Nylander et al., 1993; Gemmell et al., 1994; Tonetti, 1997). E-selectin is required for neutrophil migration from the highly vascularized gingival tissue, IL-8 is a key neutrophil chemo-attractant produced by epithelial cells, and ICAM facilitates adhesion of neutrophils to the tissue allowing formation of this wall (Springer, 1994; Darveau, 2009). Furthermore, the epithelium expresses low levels of a wide range of toll-like receptors (TLR's), including TLR1-TLR9 that mediate the response to a broad range of microorganisms (Sugawara et al., 2006; Mahanonda and Pichyangkul, 2007; Beklen et al., 2008). The array of different TLRs in combination with the multitude of bacterial species lead to a large variety of cytokines that are expressed at health (Kumar et al., 2011; Matthews et al., 2013). Studies in germ-free mice show that there are low levels of innate host mediators, such as IL-1B, present in the periodontal tissue (Dixon et al., 2004). This indicates that a basic level of cytokine expression is genetically programmed without bacterial challenge. The composition and amount of bacteria in plaque modifies cytokine expression further (Dixon et al., 2004; Kumar et al., 2011; Matthews et al., 2013).

Evidence was found of three major KPH mechanisms of *P. gingivalis* that could impair the above mentioned host defenses: (1) Toll-like receptor (TLR) response manipulation, (2) interleukin 8 (IL-8) subversion and (3) the corruption of the complement system (reviewed by Darveau, 2009, 2010; Hajishengallis and Lambris, 2011; Darveau et al., 2012).

*In vitro*, the TLR response is manipulated by *P. gingivalis* with the help of two types of lipopolysaccharides (LPS) with different lipid A structures—PgLPS1690 (type I) and PgLPS1435/1449 (type II). Type I is a TLR4 agonist thus activating the immune system, while Type II is a TLR4 antagonist inhibiting the immune response to *P. gingivalis* (Coats et al., 2005, 2007). The concentration of iron determines which type of LPS is expressed (Hanioka et al., 1990, 1991; Olczak et al., 2005). In the oral cavity, the main source of iron is hemin, found in the gingival crevicular fluid (GCF). During inflammatory process, GCF increases indicating that *P. gingivalis* type II LPS expression increases which reduces the TLR4 response. It was proposed that this could facilitate survival and multiplication of the entire microbial community (reviewed by Darveau, 2010).

*Porphyromonas gingivalis* can block production of IL-8 *in vitro*, which is produced by gingival epithelial cells in response to other bacteria, by secreting a serine phosphatase that inhibits the synthesis of IL-8 (Darveau et al., 1998; Hasegawa et al., 2008). This process is called “local chemokine paralysis” and delays the recruitment of neutrophils preventing proper neutrophil wall formation, of which was proposed that it could facilitate initial microbial colonization of the periodontium (Madianos et al., 1997; Darveau et al., 1998). Other “red complex” bacteria such as *T. denticola*, are also able to manipulate the IL-8 response of the host however the mechanism(s) involved is not understood (Ji et al., 2007).

The third and best *in vivo* documented keystone pathogen mechanism is the interference with the complement system (Hajishengallis et al., 2011; Abe et al., 2012). The complement system is a major component of the innate immune response involved in recognizing and destroying microorganisms (Walport, 2001) with complex roles in homeostasis and disease (Ricklin et al., 2010). To be a successful pathogen in humans (and any other mammal) a microorganism needs to be able to avoid complement-mediated detection and killing. Again, the best-studied example in the oral cavity is *P. gingivalis* that produces membrane bound and soluble arginine-specific cysteine proteinases called “gingipains” (Imamura, 2003). Gingipains can cleave complement factors C3 and C5 into active fragments C5a (cell activator) and C3b (phagocytosis enhancer). These fragments can be further degraded by gingipains resulting in loss of their function (Wingrove et al., 1992). However, this takes up to 1 h when adding purified compounds together *in vitro*. More relevant is that in the presence of gingipains the levels of the inflammatory mediator C5a increase within seconds (reviewed by Hajishengallis et al., 2012). This leads to an increased activation of the C5a receptor (C5aR) on leukocytes. C5aR is involved in crosstalk with TLR2, which is activated in parallel by *P. gingivalis* (and other bacterial) surface ligands. While this crosstalk leads to increased inflammation, it impairs the killing capacity for leukocytes (Wang et al., 2010; Liang et al., 2011). In mouse models this mechanism has a major role in accelerating periodontitis development and bone loss (Liang et al., 2011). A *P. gingivalis* strain that lacks gingipains failed to change the oral microbiota and induce bone loss (Liang et al., 2011). Additionally, periodontitis did not develop in mice lacking one of the two involved receptors—C5aR or TLR2 (Hajishengallis et al., 2011; Liang et al., 2011). This provides clear evidence that in mice the dysbiosis caused by *P. gingivalis* is mainly due to complement subversion.

In conclusion, it was proposed that currently known and unknown keystone pathogens use a combination of these and presently unknown mechanisms to manipulate the innate defense system leading to destructive periodontitis (Darveau, 2010).

## Applicability of the plaque hypotheses to the development of oral diseases

### Caries and ecological plaque hypothesis

**Figure 1:**
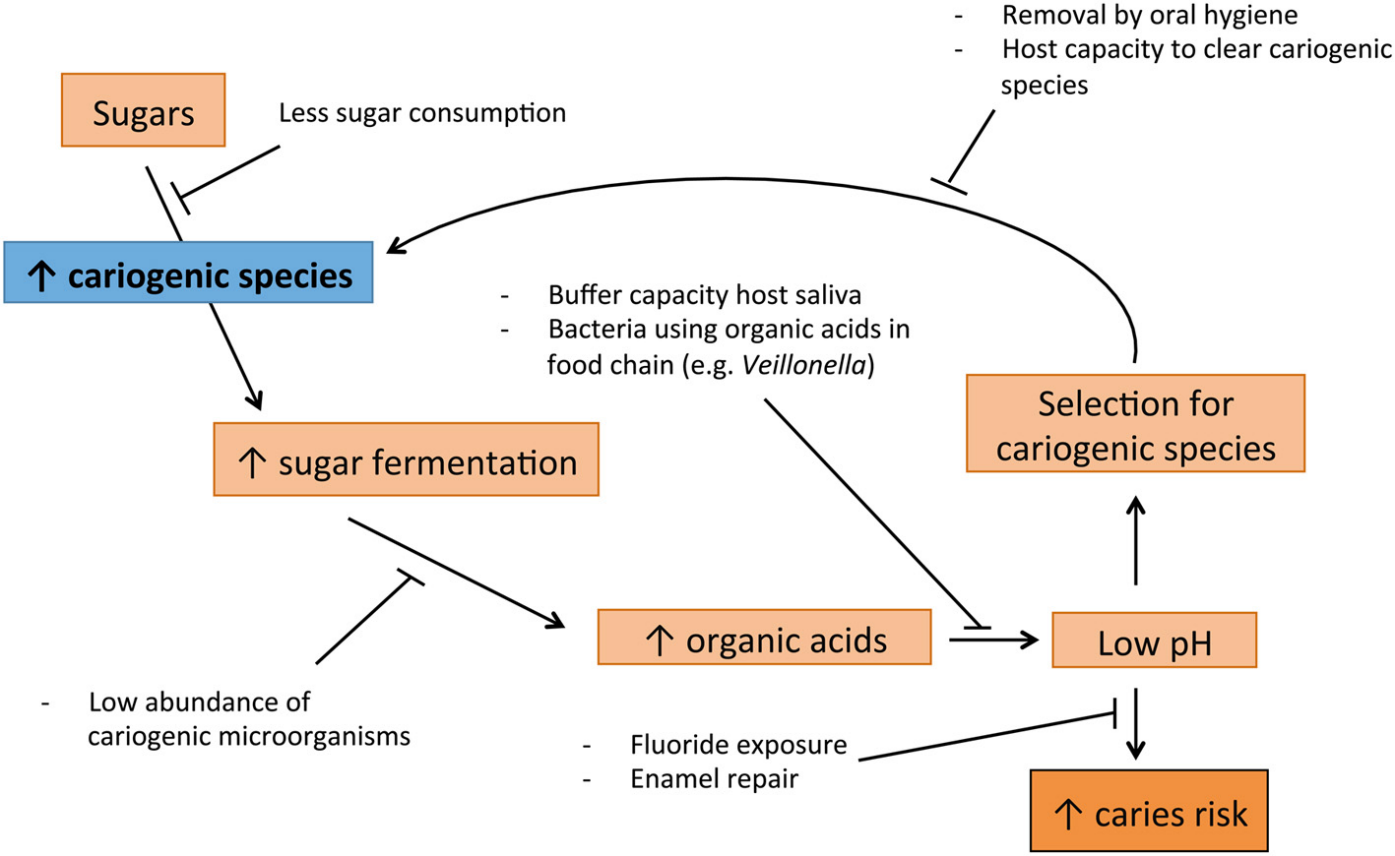
**Suggested cycle of disease leading to caries, supported by Ecological Plaque hypothesis (EPH)**.

Dental caries is a multifactorial disease, greatly influenced by the diet of the host (Touger-Decker and van Loveren, 2003). Therefore, this process fits EPH well (Figure 1): Subjects that frequently consume a considerable amount of fermentable carbohydrates, select for bacteria that ferment these carbohydrates and produce acids (Bradshaw and Marsh, 1998; Marsh, 2003). This leads to more sugar fermentation and thus acid production, increasing the cariogenic bacteria even more (Bradshaw and Marsh, 1998; Welin et al., 2003). Acidogenic (acid-producing) and aciduric (acid-tolerating) bacteria such as the classic *S. mutans, S. sobrinus* and *Lactobacillus* spp., and the later discovered *Bifidobacterium* spp., lower the pH to levels at which enamel is demineralized, which can result in caries (Loesche, 1986; Becker et al., 2002; Beighton et al., 2010). The EPH is supported also by the caries-protective role of the host-related factors such as salivary properties and fluoride exposure (Marsh and Martin, 2009).

Acid stress is a well-known biological stress factor (ergo a selective pressure) and therefore, fermentable carbohydrates are the type of nutrition that has the highest impact on the ecology of the mouth (Touger-Decker and van Loveren, 2003). Studies have shown that it is the acidic pH caused by the sugar fermentation, and not the availability of sugar itself, that leads to the disturbance of microbial homeostasis associated with caries (Bradshaw and Marsh, 1998). The aciduric bacteria are able to proliferate at acidic pH. For instance, *S. mutans* up-regulates a number of specific proteins when exposed to an acidic pH, which enhance the chances of survival under these conditions (Welin et al., 2003). In contrast, some bacteria associated with oral health are sensitive to acidic pH and are thus outcompeted in individuals that regularly consume fermentable sugars (Marsh, 2003).

*S. mutans* has carried the role of the main caries pathogen for decades (Banas, 2009) which, as discussed above, resulted in the SPH. However, demineralization can take place without the presence of this specific species, as non-mutans streptococci (e.g., *S. oralis* and *S. mitis biovar*), can metabolize sugars in comparable way to *S. mutan*s (De Soet et al., 2000). It has also been shown that phenotypic heterogeneity among different *S. mutans* strains determines the rate of the carbohydrate fermentation and thus their cariogenic potential (Burne et al., 2012).

## Periodontal diseases and the fusion of non-specific, ecological and keystone pathogen hypotheses

**Figure 2:**
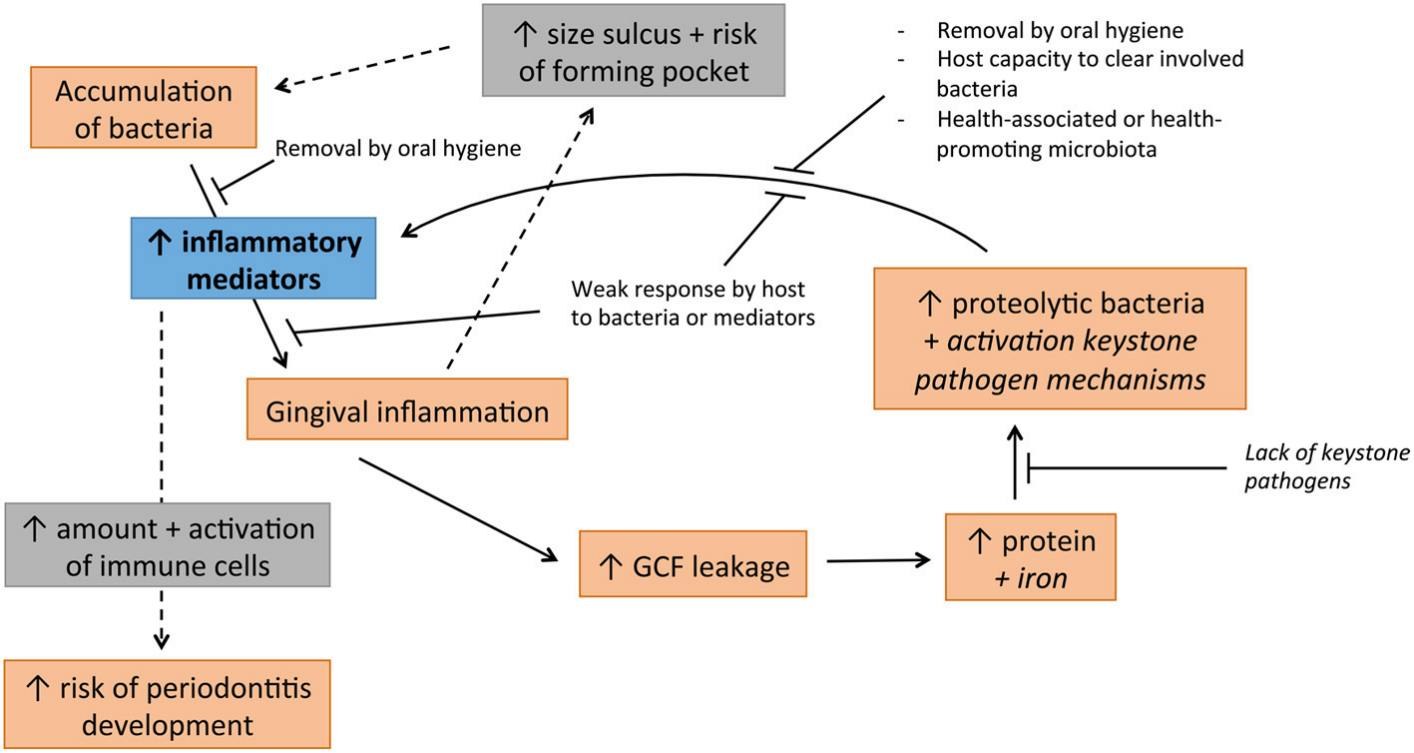
**Cycle of disease involved in development of periodontal diseases, jointly supported by non-specific, ecological and keystone pathogen hypotheses**. Italised text indicates hypothetical involvement.

Unlike caries, periodontal diseases—gingivitis and periodontitis—do not fit to a single hypothesis. The intimate interaction of bacteria with the host leading to inflammatory reaction adds to the complexity of these diseases (Figure 2).

The U-NSPH can partly explain the development of gingivitis. If plaque is allowed to accumulate without intervention by oral hygiene methods, gingivitis is established without exceptions, generally after a period of 2–3 weeks (Loe et al., 1965; Theilade et al., 1966). The finding that many members of the TLR family, including TLR1-TLR9, are present in the gingival tissue (Sugawara et al., 2006; Mahanonda and Pichyangkul, 2007; Beklen et al., 2008) has brought an old thought into reconsideration: “any microbial colonization of sufficient quantity in the gingival crevice causes at least gingivitis” (Theilade, 1986). With such a broad variety of TLRs most commensal species should be able to trigger inflammation. A broad range of bacterial products can be recognized including DNA, flagella and fimbriae, peptidoglycan and lipoteichoic acids, and LPS. Another point supporting the idea that all bacteria have a role in the gingival inflammation is that Gram-negative rods and spirochetes are not able to form plaque without Gram-positive species (Slots and Gibbons, 1978). Thus, many, if not all, species are probably directly or indirectly involved in triggering the early stages of gingivitis.

The ecological changes as indicated by the EPH are also relevant in gingivitis development. Health-associated species with an insignificant role in gingival inflammation could significantly contribute to a change in growth conditions favoring pro-inflammatory bacteria. For instance, facultative anaerobic *Rothia* spp. have recently been associated with oral health (Griffen et al., 2012; Abusleme et al., 2013; Kistler et al., 2013) and reduce the oxygen levels in the direct environment. This in turn allows proliferation of strict anaerobes, which include proteolytic Gram-negative bacteria that contribute to triggering the inflammation. The same holds for facultative anaerobic *Streptococcus* spp. that dominate the plaque in relative levels in health but decrease in disease (Matthews et al., 2013). As gingivitis develops, inflammation, gingival bleeding on probing (BoP) and the volume of GCF also increases (Kistler et al., 2013; Matthews et al., 2013). Accumulation of commensal microbiota results in an increase in GCF that in turn changes the environment because GCF contains high levels of proteins that are a novel source of nutrients (Marsh, 2003). Furthermore, GCF contains iron that triggers keystone pathogen mechanisms in *P. gingivalis* (and perhaps other bacteria) decreasing TLR4 activity *in vitro*, which could enhance survival for the whole community. This could lead to a protein-rich environment with a decreased innate response in which some bacteria can evade the immune system and are able to accumulate. Increased bacterial accumulation triggers more inflammation, which leads to a vicious circle where the host is producing more GCF, more protein and more iron (Figure 2).

Periodontitis results from complex interactions of micro-organisms and the immune system (Sanz et al., 2011). Due to increased plaque amount and increased abundance of more virulent and keystone pathogen bacteria, the concentration of inflammatory mediators increases (reviewed by Graves, 2008; Darveau, 2010). An increase in the concentration of pro-inflammatory cytokines in periodontal tissue can directly affect bone loss (Nagasawa et al., 2007).

It should be taken into account that there are differences between susceptibility for oral disease among people even if they share the same lifestyle. This could be due to the different proportions of bacterial species at health, determined by genetic factors. In recent a pyrosequencing study, subgingival plaque composition of 192 people belonging to four ethnic affiliations: non-Hispanic blacks, non-Hispanic-whites, Chinese, and Latinos, was determined. Subgingival plaque composition differed significantly between these four ethnicities, a difference that, according to the authors, was not dependent on diet and cultural differences (Mason et al., 2013). Although African Americans and Caucasians shared similar environmental factors over several generations they had different plaque compositions. Even though the authors did not provide any data to prove the consistence of environmental factors over several generations, they were able to use their results to predict ethnicity based on the dental plaque composition of random subjects. This indicates that genetic factors could have an influence in determining the core microbiome (nature over nurture), just like the ethnic background determines susceptibility for diseases such as cholera (Levine et al., 1979), pneumonia (Salnikova et al., 2013) and cystic fibrosis (Kilpatrick, 2002). These results suggest that—after controlling for socioeconomic, dietary, and other environmental factors—susceptibility for periodontal disease and caries varies among ethnicities (Cruz et al., 2009; Mason et al., 2013). Proteins involved in innate immune responses to bacterial virulence factors, e.g., TLR4 and heat shock proteins, also vary among ethnic groups (Nguyen et al., 2004; Miller and Cappuccio, 2007). This indicates that genetic host-factors have an important role in health and the shift to disease. For example, people with high expression of complement factor C5 might be more susceptible to disease progression by *P. gingivalis*-produced gingipains (*vide supra*). The role of the host in periodontal disease development should not be neglected. In transgenic mice overexpressing IL-1α, periodontitis developed even in the absence of a significant bacterial challenge (Dayan et al., 2004). In another study, it was found that aging-associated periodontitis was accompanied by lower expression of Del-1, an endogenous inhibitor of neutrophil adhesion (Eskan et al., 2012). Young Del-1-deficient mice had excessive neutrophil infiltration and developed spontaneous periodontitis. This was prevented by local administration of Del-1 which inhibited neutrophil accumulation and bone loss. It is therefore not unimaginable that periodontal disease develops in individuals with a defective immune homeostasis. For instance, individuals with defective IL-1α or Del-1 regulation could develop periodontal disease with bacterial profiles that would maintain health in other individuals. This is supported by a recent study in 385 individuals, which concluded that a single nucleotide polymorphism (SNP) in *IL-1α* was associated with periodontitis (Laine et al., 2013).

“Red complex” bacteria are in general strongly associated with periodontitis. However, there are examples of studies in which the “red complex” bacteria are below the detection threshold at diseased sites. At the same time, “red complex” bacteria can be frequently detected, be it in low numbers, in healthy sites (Socransky et al., 1998; Bik et al., 2010). Periodontitis is always been preceded by gingivitis. However, there are people with gingivitis for a lifetime who do not develop periodontitis, while others encounter rapid progression into periodontitis after only a short gingivitis episode. Apart from the genetic host factors discussed above, these findings could also be related to microbial phenotypic heterogeneity and plasticity as suggested previously (Burne et al., 2012). The role of both the host genetic background and the microbial phenotypic heterogeneity, is illustrated in a recent study by Haubek et al. (2008). *Aggregatibacter actinomycetemcomitans* is a Gram-negative rod that expresses a leucotoxin that specifically lyses human neutrophils. Compared to other strains, *A. actinomycetemcomitans* JP2 has several genetic differences (Haubek et al., 2008). As a consequence of a deletion of 530 base pairs in the promoter of the gene encoding leucotoxin, the JP2 clone produces significantly higher levels of leucotoxin. In a study in Moroccan adolescents, individuals that carried the JP2 clone had a higher risk of developing periodontitis (relative risk 18.0 vs. 3.0) (Haubek et al., 2008). Population genetic analysis suggested that, despite the global presence of the JP2 clone, it is strongly associated with the West African ethnicity thus indicating a significant host tropism effect (Haubek et al., 2008).

## All these hypotheses, but are we there yet?

The key to oral health has been described as having a diverse microbiome with two main characteristics (Zarco et al., 2012). Firstly, it should practice *commensalism within itself*, meaning that bacteria in the microbiome benefit from others without affecting them (Mikx and van Der Hoeven, 1975). Secondly, the microbiome should practice *mutualism with its host*, meaning that there is a relationship in which both partners benefit. On the one hand, the host provides nutrients and a protective environment for the microbiome. On the other hand, the microbiome contributes to the host physiology and defenses against pathogens (Zarco et al., 2012). A healthy microbiome is maintained by bacterial homeostasis which is achieved by a balance of inter-microbial as well as host-microbial interactions, which can be synergistic and antagonistic (Marsh, 1994, 2003). In this respect it should be noted that although other inhabitants of the oral cavity, including archaea, protozoa, viruses and fungi, might have significant roles in health and disease (Krom et al., 2014), most common studies on the oral microbiota are limited to bacteria. The role of these “co-inhabitants” is not well explored and thus also lacking in the above-listed hypotheses.

For the caries process, the best-fitting EPH does not consider host genetic components at all. This is striking in light of the most-unethical experiment in the history of cariology, performed in the 1950's in Sweden, known as the Vipeholm study. Mentally disabled, institutionalized individuals received high frequency carbohydrate snacks for a period of 2 years and caries incidence was scored. Depending on snack type and frequency, high levels of caries developed with the exception of about 20% of the individuals, who did not develop dental caries even at these highly cariogenic conditions (Vieira, 2012). A genetic sensitivity to caries was further supported by the observation that the parents and siblings of these individuals showed lower caries prevalence than the rest of the population (Böök and Grahnén, 1953). Since then, more and more evidence has been delivered that supports genetic susceptibility to caries (Werneck et al., 2010; Wang et al., 2013) and should be implemented in explaining the disease development.

With the KPH, the periodontal diseases, especially periodontitis, heavily depend on a single periodontal bacterium—*P. gingivalis*. This is probably due the relative ease of cultivation and genetic modification compared to the other species (Darveau et al., 2012) combined with the, by definition, low relative abundance of keystone pathogens. Other species might be equally or even more active in the process that leads from periodontal health to disease and should be investigated. Besides, it should be noted that the “keystone pathogenesis” itself has yet to be demonstrated in humans.

The large number of microbiome studies that are appearing provide a wealth of information at the taxonomic (OUT) or species level. However, in light of the known phenotypic plasticity and heterogeneity it is important to study strain differences. Although differences in bacterial phenotypes are reflected in the discussed hypotheses, mechanisms that govern them are not. The differences at strain-level rather than species level, among patients with a lifetime of gingivitis and patients that develop periodontitis rapidly should be investigated in more detail. Also, micro-evolution within the oral cavity, in which bacterial traits can be exchanged, deleted and changed, from birth to death of an individual is not covered. The host-to-host, species-to-species and strain-to-strain differences all play a role in the functioning of the oral microbiome and the way it handles environmental changes related to the ease in which it triggers disease. So far it is not known if there are fixed patterns in the shift from health to disease among people with different genetic backgrounds. The virulence of certain species or strains could differ enormously among different ethnic groups and individuals.

The recently described polymicrobial synergy and dysbiosis (PSD) model for periodontitis highlights the importance of other bacteria in keystone pathogenesis and the thought that other than the classical “red complex” species could have similar keystone roles in periodontitis (Hajishengallis and Lamont, 2012). It states that in periodontitis polymicrobial synergy can lead to dysbiosis. In this model, there are more ways to skin a cat, since different members or different gene combinations can result in a disease-provoking microbiota. In another very recent review, the importance of bacteria acting upstream and downstream of *P. gingivalis* pathogenesis is further described (Hajishengallis and Lamont, 2014). Our idea that all bacteria could have a role in periodontal disease development is supported by the PSD model which states that “traditional concepts of pathogen and commensal have become obsolete” (Hajishengallis and Lamont, 2014). For example, *S. gordonii* (commensal) can act as an accessory pathogen by increasing the virulence of *P. gingivalis*. In another very recent review applying the PSD model, the conclusion is that the transition to periodontitis requires a dysbiotic microbiota and a susceptible host (Hajishengallis, 2014). We agree that this might be true. In a Sri Lankan population with no oral hygiene habits nor dental care at all, the majority of the population (89%) experienced periodontal breakdown (Löe et al., 1986). However, 11% of this group did not have any periodontal breakdown beyond gingivitis. It is highly probable that the long-term accumulated plaque in these “periodontitis-resistant” individuals contained many late colonizing bacteria associated with periodontitis development (Kolenbrander et al., 2010). However, further research should be performed to confirm this and to conclude if it were host factors (innate immune system), bacterial factors (metagenome activity) or both functioning in a way that kept these individuals periodontitis free under conditions that would cause tissue break-down in the average person. In our opinion, the PSD-model is currently the most extensive, however it is modeled only for periodontitis.

All presently available hypotheses fall short of combining actual microbial and host behavior that lead to maintenance of health or the shift to disease. An all-encompassing hypothesis is needed, but this is only possible when sufficient knowledge is obtained between the complex relationships of the oral microbiome and the hosts' innate immune system. The advancement of technology for sequencing allows detailed analysis of the meta-genome (all potentially expressed host and microbial functions) and meta-transcriptome (all actually expressed host and microbial functions). Combined with increased computational power and more advanced bioinformatics technology, future studies will provide a more holistic view of the oral ecology and lead to unraveling of mechanisms that govern change from health to disease.

### Conflict of interest statement

The authors declare that the research was conducted in the absence of any commercial or financial relationships that could be construed as a potential conflict of interest.
